# Comparison of Cylindrical and Tapered Stem Designs for Femoral Revision Hip Arthroplasty

**DOI:** 10.3390/jcm13061745

**Published:** 2024-03-18

**Authors:** José María Hernández-Mateo, Javier Orozco-Martínez, José Antonio Matas-Díaz, Francisco Javier Vaquero, Pablo Sanz-Ruiz

**Affiliations:** 1 Department of Orthopaedic Surgery, General University Hospital Gregorio Marañón, 28007 Madrid, Spain; josehermat@gmail.com (J.M.H.-M.); javier.orozco@salud.madrid.org (J.O.-M.); jvaquero@salud.madrid.org (F.J.V.); 2 Surgery Department, School of Medicine, Complutense University of Madrid, 28040 Madrid, Spain

**Keywords:** femoral revision, cylindrical stems, tapered stems

## Abstract

**Background:** Cylindrical fully-coated cobalt-chromium stems (CCS) have been widely used in femoral revisions. However, monoblock fluted conical tapered stems (FCTS) are growing in popularity. The present study seeks to determine whether there are any long-term differences between the two designs. **Material and methods:** A retrospective study of 38 CCS versus 40 FCTS was carried out. Demographic data, clinical variables and radiographic parameters were recorded. **Results:** Demographic data were comparable. A greater proportion of septic revisions, periprosthetic fractures and previous osteosynthesis failures was observed with FCTS versus CCS (*p* = 0.012). A greater use of FCTS was recorded in cases with bone defects of type IIIA and higher (*p* = 0.025). There were no significant differences in terms of in-hospital complications (*p* = 0.815), postoperative surgical complications or need for reoperation (*p* = 0.156). The CCS group presented a higher percentage of clinical thigh pain at the end of follow-up (*p* = 0.006). Additionally, a greater presence of radiolucencies was observed with CCS, especially in proximal zones (1, 7, 10 and 14). More subsidence, tip cortical hypertrophy and stress shielding were recorded in the CCS group. The overall survival at 120 months was 84.2% in the CCS group and 85% in the FCTS group (*p* = 0.520). When analyzing isolated aseptic loosening as the cause of failure, the survival rate was 94.7% in the CCS group and 95% in the FCTS group (*p* = 0.506). **Conclusions:** Both FCTS and CCS with diaphyseal anchorage afford excellent long-term survival rates, with no differences between the two designs. However, a higher incidence of stress shielding, radiolucencies and thigh pain with CCS seems to favor the use of FCTS.

## 1. Introduction

The frequency of revision hip replacement is increasing due to growing number of primary arthroplasties, aging of the population, and the complexity of some primary total hip arthroplasties [1]. Likewise, a parallel rise is observed in the number of revisions performed in the same patient, with a dramatic increase in their complexity, associated with greater bone defects [2].

Cementless stems have shown the best medium- and long-term survival outcomes in femoral revision [3,4,5,6,7,8,9,10] compared with cemented stems due to the difficulty in achieving adequate bone cement interdigitation in a sclerotic bone or in the cortical diaphysis itself, except when used in conjunction with the impaction grafting technique.

Porous fully-coated cobalt-chromium stems (CCS) have been widely used in femoral revisions. However, in the last 20 years, fluted conical tapered stems (FCTS) have grown in popularity [4,11,12]. While CCS have shown excellent long-term survival outcomes across various types of bone defects and have been the predominant choice for many years [5], they carry a risk of mechanical failure attributed to inadequate distal fixation in more severe femoral bone defects, as well as the stress shielding effect associated with their usage. Moreover, they are linked to a higher incidence of complications in severe type IIIB and IV femoral defects, particularly when longer stem lengths and larger diameters are necessary [13], hence the importance of analyzing femoral component revisions and thus attempting subgroup analysis. This has led to an increased use of other types of stems, such as FCTS. These stems have ribs to improve rotational stability and a conical shape that improves the load transfer to the bone, increasing the surface area of contact. Furthermore, these stems are manufactured from titanium alloys, which are more elastic than those made of chrome-cobalt, further enhancing load transfer between the implant and the bone, and thereby facilitating osseointegration and proximal bone remodeling [4]. Following an initial period of disappointing results based on the learning curves, these stems have subsequently yielded excellent results, with lower failure rates than with the cylindrical designs in more severe femoral defects [4,14,15]. However, most series to date involve poor follow-up or combine different stem types in each group.

The present study seeks to determine whether there are any long-term differences between the two designs in terms of implant survival, radiological findings suggestive of loosening and functional outcomes. Our objectives were, firstly, to compare the survival rates of the two stem types in a group of patients with equivalent bone defects over prolonged follow-up. Secondly, we aimed to assess and compare any possible functional differences in the long-term clinical outcomes between the two implant types. Lastly, we explored whether there is a higher rate of aseptic loosening and stress shielding with the use of the more rigid stem design. To the best of our knowledge, this is the first study comparing the clinical and functional outcomes of two different stem philosophies for treating bone defects over more than 10 years of follow-up.

## 2. Materials and Methods

All patients gave informed consent before their inclusion in the study. The study was carried out on a retrospective basis, reviewing a prospectively compiled database, and was conducted in accordance with the principles of the 1964 Declaration of Helsinki (as revised in 2013). The study protocol was approved by the Research Ethics Committee of our center.

Between 2006 and 2012, a total of 395 hip prosthesis revisions were performed. Of these, 145 involved isolated revisions of the femoral component, and 87 were carried out with an uncemented monoblock stem. Those patients who did not complete a minimum follow-up of 10 years or those in which the main cause of revision was neoplastic disease were excluded from the study, thus leaving a total of 78 patients. The patients were divided into two groups according to the type of stem used: cylindrical (38 hips) or tapered (40 hips). A single prosthetic design was used for all patients in each group. A monoblock porous fully-coated cylindrical stem (CCS) made of cobalt-chromium alloy (Solution Stem^®^, DePuy^®^, Warsaw, IN, USA) was used in the cylindrical stem group, while a monoblock fluted conical tapered stem (FCTS) made of titanium alloy (Wagner SL^®^, Zimmer^®^, Warsaw, IN, USA) was used in the tapered stem group.

The surgical technique was the same in all patients, except the aspects specific to the reaming of each type of stem. The perioperative protocol was identical in both groups. In cases where no septic cause was suspected, 2 g of intravenous cefazolin was administered 30 min before the induction of anesthesia. When the cause of revision was sepsis (one time or second time replacement), teicoplanin 600 mg and meropenem 1 g were administered until the culture results were obtained, followed by de-escalation to a targeted antibiotic treatment. All patients undergoing procedures received spinal anesthesia, with general anesthesia in those procedures expected to last more than three hours. The surgical approach was posterolateral in all cases, using an extended trochanteric osteotomy (ETO) in the cases of difficult extraction of the previous stem or when deformity of the proximal femur could impede correct implantation of the new stem. In all cases, the femoral bed was prepared with rigid reams, reaming the same diameter as the definitive stem—except when it was necessary to implant a curved stem, where flexible drills with 0.5 mm over-reams were used. In all cases, the shortest stem length needed to achieve sufficient stability was implanted; in this regard, cortical contact with the stem length equal to twice the diameter of the femoral canal at the level of isthmus was considered to be sufficient. Partial weight bearing with two canes was authorized at 24–48 h according to tolerance. In the event where appropriate press-fit could not be obtained, weight bearing was delayed until 6 weeks postoperatively. All patients were reviewed regularly at 6 weeks, 3 months, 6 months and annually to assess function and perform radiographic controls.

### 2.1. Patient Assessment

Demographic data such as age, sex, operated side, body mass index (BMI), stature, comorbidities and the cause of revision surgery were recorded in all patients. Functional assessment was based on the Harris Hip Score (HHS) (preoperatively and at each follow-up visit). In addition, the degree of patient satisfaction was recorded and classified as follows: satisfied, regularly satisfied or dissatisfied. Thigh pain (“clinical peak effect”) was also recorded, as well as the need to use walking aids (one cane, two canes, a walker or a wheelchair).

During hospital stay, in addition to intraoperative data, we collected the need for ETO, bone grafting and implant size/length, preoperative and immediate postoperative (24–48 h) hemoglobin levels and the days of hospital stay. All intraoperative and immediate postoperative complications (<24 h), as well as orthopedic (acute surgical wound infection, dislocation, periprosthetic fracture, sciatic nerve palsy) or medical (urinary tract infection, pneumonia, paralytic ileus, congestive heart failure, delirium or death) complications during hospital stay were recorded. Lastly, any surgical complications during follow-up (dislocation, subacute or chronic infection, new loosening or periprosthetic fracture) and the need for further revision surgery were recorded.

### 2.2. Radiological Analysis

**Figure 1:**
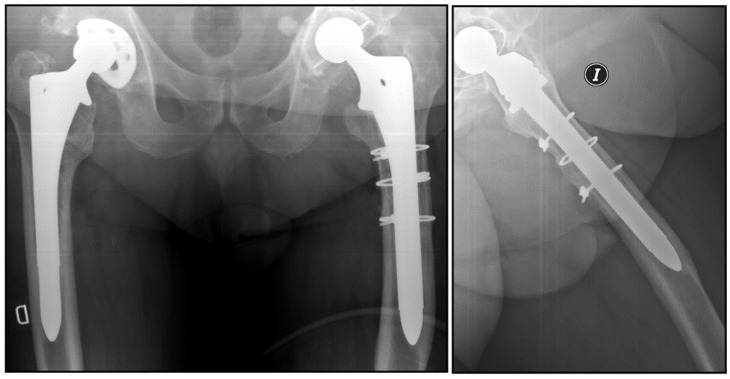
Cortical tip hypertrophy in a patient with a cylindrical stem.

In all patients, supine anteroposterior and axial radiographs were taken on the first postoperative day, at 3 months, 6 months and annually thereafter. Full weight-bearing radiographs were obtained preoperatively and one year after surgery. The type of femoral defect was assessed preoperatively according to the Paprosky classification [16]. Stress shielding was determined based on the Bohm and Bischel criteria [17]. The presence of radiolucencies larger than 2 mm was analyzed at one year and at the end of follow-up, and their location was classified according to Gruen areas. Cortical hypertrophy (Figure 1) was recorded at the level of the stem tip [18]. Possible postsurgical dysmetria was assessed by means of loaded anteroposterior teleradiographs. Stem subsidence [11] was measured according to Callaghan’s method (defined as >5 mm) [19]. These data were assessed by two orthopedic surgeons specialized in prosthetic hip revision surgery. In the case of disagreement, a third surgeon assessed the measurements.

### 2.3. Statistical Analysis

The Student *t*-test for independent samples was used for the comparison of continuous quantitative variables, in which a normal distribution was assumed, as assessed by a Shapiro–Wilk test. Pearson’s chi-square test and Fisher’s exact test were used in the case of qualitative variables. In case of statistical significance, effect measure size tests were used: Cohen’s D for quantitative variables and Phi Coefficient and Cramer’s V for qualitative variables, with a level of significance of *p* < 0.05. With regard to survival analysis, the Kaplan–Meier method was used for any cause of revision, and specifically, revisions secondary to aseptic loosening, based on the log-rank statistic. Subgroup analysis was performed according to the Paprosky type of femoral bone defect (type I, II, IIIA and IIIB), with the same variables as mentioned above. An α error < 5% (*p* < 0.05), with a β error < 20%, was considered statistically significant. The SPSS version 19.0 statistical package was used throughout.

## 3. Results

Demographic data were recorded (Table 1). No significant differences were observed between the groups, which were thus considered to be comparable. The overall mean duration of follow-up was 127.3 ± 31 months, with no significant differences between the two groups (CCS 128.2 ± 28 months vs. FCTS 126.6 ± 34 months; *p* = 0.890).

A greater proportion of septic revisions, periprosthetic fractures and previous osteosynthesis failures was recorded with FCTS versus CCS (V = 0.405; *p* = 0.012). A greater use of FCTS was observed in cases with bone defects of type IIIA and higher (27.5% CCS versus 45% FCTS; V = 0.301; *p* = 0.025). Table 2 shows the stem lengths used.

### 3.1. Clinical Results

There were no statistically significant differences between the groups in terms of the need for ETO or bone grafting. Likewise, there were no differences in blood loss or severe acute anemization requiring transfusion support (Table 3).

No significant differences were observed between the groups in terms of in-hospital complications (five CCS versus six FCTS; *p* = 0.815), postoperative surgical complications or the need for reoperation (10.5% in CCS versus 22.5% in FCTS; *p* = 0.156). However, in the case of new revision surgery, the latter was seen to be performed earlier in the FCTS group (33 months versus 80 months; d = 0.677; *p* = 0.043).

No differences were observed between the two groups regarding the HHS both at one-year (CCS = 72.5 ± 13.6 and FCTS = 67.9 ± 18.7; *p* = 0.233) and at the end of follow-up (CCS = 64.3 ± 20.1 and FCTS = 64.7 ± 19.9; *p* = 0.894). Likewise, there were no significant differences in the need for walking aids at the end of follow-up (*p* = 0.332) or in the degree of patient satisfaction (*p* = 0.686). However, patients in the CCS group had a higher percentage of clinical thigh pain at the end of follow-up (CCS = 11% versus FCTS = 2%; r_ϕ_ = 0.325; *p* = 0.006).

### 3.2. Radiographic Results

A greater presence of radiolucencies was observed with the use of CCS at one year and at the end of follow-up, particularly in proximal zones (1, 7, 10 and 14 Gruen zones) (Table 4 and Table 5).

**Figure 2:**
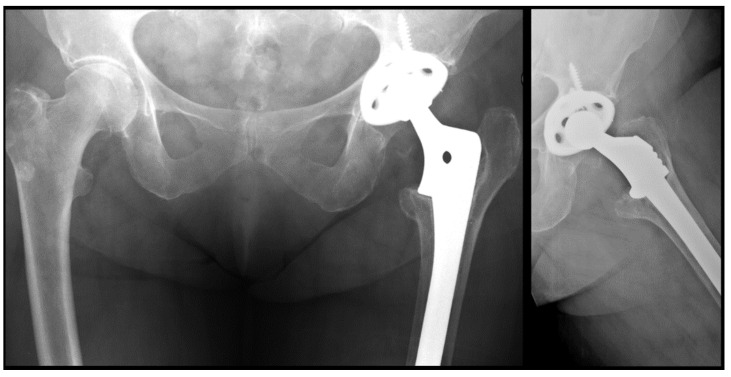
Stress shielding in a patient with a cylindrical stem.

This greater presence of radiolucencies was significantly associated with a higher incidence of stress shielding as well as with greater cortical hypertrophy at the tip of the stem (CCS = 10% versus FCTS = 3%; r_ϕ_ = 0.272; *p* = 0.032) (Figure 2). 

**Figure 3:**
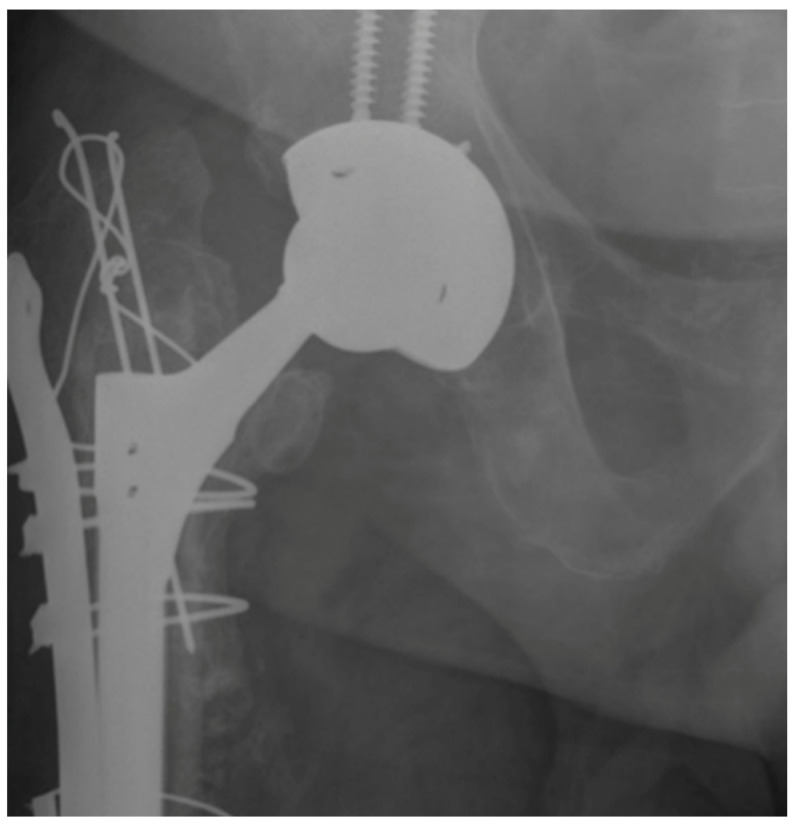
Stem subsidence in a fluted conical tapered stem.

On the other hand, the use of FCTS was associated with a higher risk of stem subsidence > 5 mm at one-year (CCS = 2% versus FCTS = 10%; r_ϕ_ = 0.266; *p* = 0.012) and at the end of follow-up (CCS = 4% versus FCTS = 14%; r_ϕ_ = 0.202; *p* = 0.03) (Figure 3). 

There were no significant differences in post-surgical dysmetria (CCS = 6.8 mm and FCTS = −12 mm; *p* = 0.576).

When analyzed by subgroups, CCS used for type II defects showed more radiolucencies in zones 1 (r_ϕ_ = 0.283; *p* = 0.004), 10 (r_ϕ_ = 0.232; *p* = 0.04) and 14 (r_ϕ_ = 0.293; *p* = 0.002) at one year of follow-up. At the end of follow-up, this finding was confirmed in zones 1 (r_ϕ_ = 0.237; *p* = 0.02), 10 (r_ϕ_ = 0.261; *p* = 0.025) and 14 (r_ϕ_ = 0.270; *p* = 0.008). In turn, there were no differences in stem subsidence (>5 mm) at one year (*p* = 0.999) and at the end of follow-up (*p* = 0.394). In relation to stress shielding, no differences were found at one year (*p* = 0.923) and at the end of follow-up (*p* = 0.204). In contrast, there were statistically significant differences in thigh pain (r_ϕ_ = 0.472; *p* = 0.03) when CCS was used for type IIIA femoral defects. These clinical findings were accompanied by radiological correlations, with differences in cortical tip hypertrophy at one year for the same subgroup (r_ϕ_ = 0.337; *p* = 0.044) and at the end of follow-up.

### 3.3. Survival Analysis

**Figure 4:**
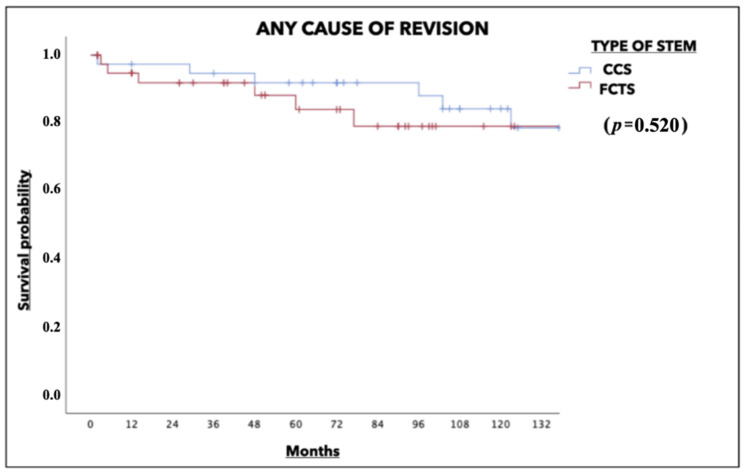
Survival rates for any cause of revision.

**Figure 5:**
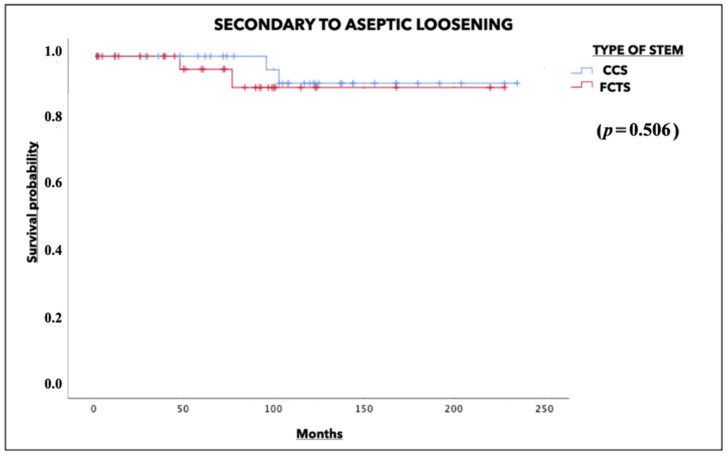
Survival rates for aseptic loosening.

The survival rate determined for any cause of revision surgery at 120 months was 84.2% in the CCS group versus 85% in the FCTS group (*p* = 0.520). When isolated aseptic loosening was analyzed as a cause of failure, the survival rate was found to be 94.7% in the CCS group and 95% in the FCTS group (*p* = 0.506) (Figure 4 and Figure 5).

## 4. Discussion

Aseptic loosening remains one of the main reasons for hip revision. Despite the excellent results published with cemented fixation in primary surgery, such fixation has not demonstrated consistent results in femoral revision if not combined with an impacted graft technique, leaving cementless stems as the best option in this type of surgery [20]. The use of CCS has shown excellent medium- and long-term outcomes, with survival rates of over 95% [13], and such implants have been the preferred option for many surgeons despite the appearance of negative phenomena typical of this stem type, such as stress shielding or an increased incidence of thigh pain. On the other hand, these femoral designs have shown higher failure rates when there is less than 4 cm of diaphyseal fixation or when it is necessary to use stems with a diameter of over 18 mm [13]; as a result, there has been an increase in the use of stems with different designs.

One of the most widely studied alternatives to CCS is the use of conical stems with flutes, made of titanium and with a Young’s modulus of elasticity closer to that of bone. The conical shape, which increases bone surface contact (especially at the proximal level), the overall implant surface and the different materials of the stem (i.e., titanium or with a less elastic modulus that is closer to the bone) have been associated with proximal bone remodeling due to higher physiological load transfer, collectively producing an increase in bone density. This is known as the Wagner effect and has been extensively described [21,22,23]. Several authors have obtained excellent results in severe bone defects with this type of stem [24,25]. However, few series [4,5,24] have compared the two stem designs in the conventional treatment of femoral defects, and the mean follow-up reported to date is no longer than 6 years. Therefore, the purpose of the present study was to compare the long-term survival of these two prosthetic designs and to establish whether the design influences the functional outcomes or whether a tapered and more elastic geometry can reduce the complications of distal rigid fixation observed with full-coverage cylindrical stems.

We observed no differences between the two prosthetic designs in terms of survival related to any cause of failure or aseptic loosening at more than 10 years of follow-up, which exhibited an overall survival rate of 85%, which is similar to that reported in other series [15,26,27,28]. In this respect, Engh et al. [28] reported a survival rate of 89% at 10 years [28]. In our series, despite a higher incidence of thigh pain and cortical hypertrophy, we observed no significant differences in terms of survival between the two designs in severe femoral defects.

The use of both prosthetic designs resulted in no differences in functional outcomes, satisfaction or the need for walking aids. However, tapered stems were more frequently used for septic revision surgeries, septic problems, periprosthetic fractures or osteosynthesis failures, which could speak in favor of employing this stem type in more complex cases, thus achieving optimal functional outcomes. On the other hand, a greater percentage of thigh pain due to tip effects was observed with CCS. The increased tip effect recorded in our study (28.9% versus 5%; clinical and radiological, translated as cortical hypertrophy) with CCS has been described in previous studies [3,4]. It was similar to the published figures (between 8–30%) and was caused by inadequate osseointegration of the implant in the diaphyseal area [20,26]. The cylindrical design, which favors distal fixation in the narrower isthmus area, as well as the greater rigidity of cobalt-chromium over titanium, seem to condition proximal bending around the stem, resulting in a “bell-shaped” effect with load transmission at the stem tip [26], which was responsible for radiological cortical hypertrophy and increased pain at that level.

The use of CCS was associated with more radiolucencies in zones 1, 7 and 14 (corresponding to the metaphyseal zone) at both one-year and at the end of follow-up. In addition, more radiolucencies were also observed in zone 10 (diaphyseal zone). These radiolucencies can be attributed to the difficulty of achieving minimal metaphyseal fixation with the use of this type of stem. In parallel to the increase in proximal radiolucencies, we observed a significantly greater percentage of stress shielding with the use of CCS. The theoretically better load transmission of tapered stems [29,30,31], together with the lower modulus of elasticity of titanium, seem to protect against this effect. The percentage of stress shielding found in cylindrical stems at the end of follow-up (67.5%) was slightly higher than that reported in other series, such as that of Zhang et al. [4] (38.8%) or Richards et al. [5] (41.9%). This may be due to the fact that the mean duration of follow-up in our series was longer than in the rest of publications.

While biomechanical studies have reported that tapered stems require greater loads to produce implant subsidence [30], other authors [18,27] have concluded that tapered stems exhibit greater subsidence, as measured in millimeters, in the first year compared with cylindrical stems, given the difficulty of achieving adequate diaphysis filling with long straight stems when used for defects of type IIIA or more—obtaining a three-point anchorage due to femoral curvature, which conditions loss of the theoretical biomechanical superiority of these designs. These statements agree with the data obtained in our study, where greater subsidence was observed with FCTS one year after surgery, followed by stabilization after this time. This was the main reason why revisions in the FCTS group were made earlier than in the CCS group.

In our series, the number of intraoperative fractures in the CCS group was no greater than reported in previous studies [5], probably due to the over-milling performed when curved stems were used. The complication rates with the two stems were similar, and in line with the published literature, with an incidence of approximately 5–20% in both groups [4,5,26]. However, the previous greater rate of revision surgery due to instability observed in the FCTS group is significant. The increased risk of dislocation with the use of these stem designs has been previously noted as a consequence of mistakes in early designs [32,33] and the increased risk of initial postoperative subsidence [4,5,26,34].

The present study has some limitations. Firstly, the retrospective, non-randomized nature of the study requires caution in drawing conclusions and extrapolating results. Nevertheless, the use of a single prosthetic design per group, the homogeneity of the surgeries (all performed by a small group of prosthetic surgeons, with the same surgical approach and the same perioperative protocol) and the long follow-up period involved in both groups reinforce the conclusions obtained. Secondly, conclusions are limited to the stems used and cannot be generalized to other designs. The use of different alloys for the manufacture of stems has a direct bearing on the results obtained with them, and not everything can be summed up in their external geometry. Thirdly, the sample size involved does not allow for analysis between subgroups. However, the aim of this study was to assess differences between the two stem types in common situations of femoral bone defects, not only in a specific type of bone defect—especially considering that, in severe bone defects, different series have reported superior results with the use of modular stems. Lastly, and despite no previous evidence in this regard, the smaller average stature of the population in our country compared to the populations in other series (American, Canadian and English) could justify obtaining a slightly more distal diaphyseal anchorage and an increase in proximal radiolucencies without affecting the implant survival rate in our series.

## 5. Conclusions

In summary, the present study suggests that tapered and cylindrical monoblock stems with diaphyseal anchorage afford excellent long-term results in terms of survival and function, with no significant differences between them. However, the greater incidence of stress shielding, radiolucencies and thigh pain in the cylindrical stem group seems to favor the use of tapered stems.

## Figures and Tables

**Table 1:** Demographic data.

Demographic Data	Cylindrical Stems (N = 38)	Tapered Stems (N = 40)
Age (Years)	68.55 ± 12.78	71.38 ± 12.86
Gender (Male/Female)	21/17	21/19
Side (Right/Left)	15/23	15/25
BMI ^1^ (kg/m^2^)	26.54 ± 5.21	26.93 ± 4.97
Stature (cm)	164.68 ± 6.73	167.33 ± 5.22
Smokers (%)	28.9%	17.24%
Presence of DM (%)	10.5%	24.1%
Heart failure (%)	18.42%	20.7%
CKD ^2^ (%)	21.1%	10.3%
Immunodeficiency (%)	2.6%	5.2%
Reasons of revision (n)		
Aseptic loosening	31	20
Infection	5	5
Periprosthetic fracture	2	6
Osteosynthesis failure	0	5
Recurrent dislocation	0	1
Two-stage revision (n)	2	3
Pre-surgical HHS Jr ^3^	47.4 ± 15.3	49.3 ± 13.9
Paprosky femoral defect (n)		
Type I	5	0
Type II	21	20
Type IIIA	11	18
Type IIIB	1	2
Acetabular revision (%)	42.1%	62.1%
Bone graft use (%)	42.1%	48.3%
Femoral osteotomy (n)		
Not needed	19	27
Trochanteric	8	2
ETO ^4^	11	11

^1^ Body mass index. ^2^ Chronic kidney disease. ^3^ Harris Hip Score Junior. ^4^ Extended trochanteric osteotomy.

**Table 2:** Lengths of stem used.

Lengths of the Stem Used (n)	
**FCTS group (n = 40)**	
190 mm	2 (5%)
225 mm	11 (27.5%)
265 mm	15 (37.5%)
305 mm	11 (27.5%)
345 mm	1 (2.5%)
**CCS group (n = 38)**	
203 mm	17 (44.7%)
254 mm	21 (55.3%)

**Table 3:** Clinical results.

Clinical Results	CNCCS (N = 38)	TNTS (N = 40)	Signification
Length of stay	19.9 ± 2.1	21.5 ± 2.1	*p* = 0.586
Blood loss (g/dL)	4.7 ± 1.5	4.4 ± 1.1	*p* = 0.459
Bone grafting (n)	16 (42.1%)	19 (47.5%)	*p* = 0.632
Need for ETO (n)	11 (28.9%)	11 (27.5%)	*p* = 0.840
2-stage revision (n)	2 (5.3%)	3 (7.5%)	*p* = 0.687
Intraoperative periprosthetic fracture (n)	6 (15.7%)	8 (20%)	*p* = 0.535
Post-op complications (n)			*p* = 0.658
Dislocation	3 (7.9%)	3 (7.5%)	
Infection	1 (2.6%)	3 (7.5%)	
Periprosthetic fracture	0	1 (2.5%)	
New loosening	3 (7.9%)	3 (7.5%)	
Patient satisfaction (n)			*p* = 0.247
Unsatisfied	6	11	
Fairly satisfied	12	7	
Completely satisfied	20	21	

**Table 4:** Radiolucencies by Gruen zones at a year of follow-up.

Radiolucencies by Gruen Zones at a Year of Follow-Up (n)	CNCCS (n = 38)	TNTS (n = 40)	Signification
Zone 1	14	5	*p* = 0.012 *
Zone 2	4	2	*p* = 0.360
Zone 3	5	1	*p* = 0.077
Zone 4	5	1	*p* = 0.077
Zone 5	5	1	*p* = 0.077
Zone 6	3	2	*p* = 0.602
Zone 7	10	3	*p* = 0.026 *
Zone 8	7	3	*p* = 0.149
Zone 9	4	1	*p* = 0.149
Zone 10	6	1	*p* = 0.040 *
Zone 11	3	1	*p* = 0.280
Zone 12	3	1	*p* = 0.280
Zone 13	4	2	*p* = 0.360
Zone 14	13	4	*p* = 0.010 *

* Significative differences was observed.

**Table 5:** Radiolucencies by Gruen zones at the end of follow-up.

Radiolucencies by Gruen Zones at the End of Follow-Up (n)	CNCCS (n = 38)	TNTS (n = 40)	Signification
Zone 1	15	7	*p* = 0.042 *
Zone 2	6	5	*p* = 0.744
Zone 3	6	3	*p* = 0.286
Zone 4	5	2	*p* = 0.233
Zone 5	6	2	*p* = 0.134
Zone 6	4	3	*p* = 0.691
Zone 7	12	4	*p* = 0.024 *
Zone 8	9	4	*p* = 0.127
Zone 9	7	2	*p* = 0.075
Zone 10	7	1	*p* = 0.025 *
Zone 11	4	1	*p* = 0.165
Zone 12	3	2	*p* = 0.643
Zone 13	4	5	*p* = 0.722
Zone 14	15	6	*p* = 0.020 *

* Significative differences was observed.

## Data Availability

Data is unavailable due to privacy restrictions.
